# Sarcoidosis-Induced Hypercalcemia in a Patient With Multiple Endocrine Neoplasia Type 2A (MEN2A) Syndrome Harboring the C609Y REarranged During Transfection (RET) Mutation

**DOI:** 10.1016/j.aed.2026.01.012

**Published:** 2026-01-30

**Authors:** Eiman Ibrahim, Zarish Cheema, Praveen Walaliyadda, Muhammad Moseeb Ali Hashim, Mohamed Elbanan, Uzma Khan

**Affiliations:** 1 Department of Endocrinology, University of Missouri School of Medicine, Columbia, Missouri; 2 Department of Rheumatology, University of Missouri School of Medicine, Columbia, Missouri; 3 Department of Pathology, University of Missouri School of Medicine, Columbia, Missouri; 4 Department of Radiology, University of Missouri School of Medicine, Columbia, Missouri

**Keywords:** genetic syndromes, hypercalcemia, hyperparathyroidism, multiple endocrine neoplasia, sarcoidosis

## Abstract

**Background/Objective:**

Hypercalcemia is a common clinical manifestation of Multiple Endocrine Neoplasia 2A (MEN 2A) through primary hyperparathyroidism.

The objective of this report is to describe a rare and unique case of sarcoidosis-related hypercalcemia in a patient with a history of MEN2A.

**Case Report:**

A 59-year-old female with past medical history of MEN2A, REarranged during Transfection mutation C609Y, diagnosed in 2007 status post-total thyroidectomy presented to the hospital with an acute abdominal pain aggravated with eating. Review of systems was significant for a 40 lbs. weight loss, fatigue, dry mouth, and polydipsia. Computed tomography (CT) abdomen showed liver and spleen nodules and a few mildly enlarged retroperitoneal lymph nodes. CT chest noted enlarged left axillary lymph nodes but otherwise no enlarged mediastinal lymph nodes. Blood work showed a calcium level of 13.6 [8.5-10.3 mg/dl], intact parathyroid hormone: 6.3 [10-65 pg/mL], 25-OH vitamin D level: 21.5 [18-50 ng/mL], 1,25-vitamin D: 51 [18-64 pg/mL]. Liver biopsy showed noncaseating granuloma. Positron emission tomography-computed tomography showed hypodense splenic nodules, heterogenous liver, diffuse pancreatic atrophy, and lack of abnormal neck/lung adenopathy. The patient was then referred to rheumatology for evaluation for sarcoidosis. Angiotensin-converting enzyme level was 124. Patient was managed acutely with calcitonin and was started on prednisone and hydroxychloroquine. Calcium level normalized.

**Discussion:**

The co-occurrence of both MEN 2A and sarcoidosis is rare, adding an unexpected layer of complexity to the diagnosis.

**Conclusion:**

The rarity of sarcoidosis co-occurring with MEN 2A highlights the importance of considering a broad differential diagnosis, even in patients with known genetic syndromes, to ensure accurate management.

**Highlights**

• Multiple Endocrine Neoplasia 2A (MEN 2A) with sarcoidosis raises hypercalcemia risk due to overlapping mechanisms
• Rare MEN 2A and sarcoidosis co-occurrence requires broad differential diagnosis
• Individualized multidisciplinary care is key for monitoring calcium and parathyroid hormone

**Clinical Relevance**

This case is of clinical significance as an illustration of the presentation of an unexpected cause of hypercalcemia. This highlights the need for a broad differential diagnosis to ensure accurate management and appropriate therapeutic interventions, as surgical resection is needed for hyperparathyroidism, while medical management is indicated for sarcoidosis-related hypercalcemia.

## Introduction

Multiple Endocrine Neoplasia Type 2A (MEN 2A) is a rare hereditary syndrome caused by a mutation in the REarranged during Transfection (RET) proto-oncogene and characterized by the presence of medullary thyroid carcinoma, pheochromocytoma, and primary hyperparathyroidism.^1^ Primary hyperparathyroidism (PHPT) in MEN 2A results from hyperplasia or adenomas of the parathyroid glands, leading to excessive secretion of parathyroid hormone (PTH). The increased PTH levels stimulate osteoclastic bone resorption and renal tubular reabsorption of calcium, as well as increased intestinal absorption of calcium mediated by the kidneys' conversion of vitamin D to its active form. As a result, hypercalcemia is a common clinical manifestation in patients with MEN 2A.^2^^,^^3^

The prevalence of PHPT in MEN 2A is variable across clinical studies. According to a nationwide population-based retrospective study in Denmark, the frequency of PHPT in MEN 2A was found to be 8%.^4^ In contrast, a retrospective multicentric study in France reported a higher prevalence, with PHPT occurring in 20% to 30% of individuals with MEN 2A. This discrepancy may be due to variances in study populations, diagnostic criteria, and methodologies.^5^ An important confounding factor can also be the different RET mutation; for example, PHPT individuals were affected by the Danish RET C611 founder effect.^6^ This founder effect can indeed mask the true genotype–phenotype relationships and led to misclassification of PHPT risk.

Overall, the risk of hypercalcemia and MEN2A individuals with RET C609Y mutation is significantly lower than those with mutations at other codons such as 634, which have a higher prevalence of PHPT and associated hypercalcemia.^7^^,^^8^

When a patient with MEN 2A also has sarcoidosis, the potential for hypercalcemia is intensified. Sarcoidosis is a systemic granulomatous disease in which activated macrophages within granulomas convert 25-hydroxyvitamin D to 1,25-dihydroxyvitamin D (calcitriol) independent of the regulatory feedback mechanisms typically exerted by PTH and serum calcium levels.^9^ This dysregulated production of calcitriol leads to increased intestinal absorption of calcium, contributing to hypercalcemia.^10^

In a patient with both MEN 2A and sarcoidosis, the overlapping pathophysiological mechanisms can significantly raise the risk of hypercalcemia. Management of such a patient would require a multidisciplinary approach, involving regular monitoring of serum calcium and PTH levels, as well as imaging studies and other diagnostic evaluations to assess the activity of both parathyroid glands and sarcoidosis. Therapeutic interventions may include surgical resection of hyperactive parathyroid tissue, corticosteroids to reduce granulomatous activity in sarcoidosis, and the use of medications such as bisphosphonates or calcimimetics to manage hypercalcemia. This complex interplay necessitates a careful and individualized treatment strategy to prevent complications associated with severe hypercalcemia.

Sarcoidosis leads to elevated calcium levels through increased calcitriol production by granulomas, independent of PTH.^11^ This unexpected cause of hypercalcemia in a MEN 2A patient adds diagnostic complexity, as clinicians would initially focus on parathyroid involvement. The rarity of sarcoidosis co-occurring with MEN 2A highlights the importance of considering a broad differential diagnosis, even in patients with known genetic syndromes, to ensure accurate diagnosis and management.

Here, we describe a case of non-PTH-mediated hypercalcemia due to granulomatous disease involving a relatively rare site, which raised an important diagnostic challenge.

## Case Presentation

A 59-year-old female with a history of MEN2A due to a RET proto-oncogene C609Y mutation, diagnosed in 2007 and status post-total thyroidectomy, presented to the hospital with an acute onset of abdominal pain, which was exacerbated by food intake. She had no prior history of hypercalcemia or hyperparathyroidism, and her most recent serum calcium level prior to this presentation was 9.3 mg/dL (reference range: 8.5–10.3 mg/dL), with PTH within the normal range at 28.7 pg/mL (reference range: 10–65 pg/mL). Her review of systems was notable for a 40-pound unintentional weight loss over several months, chronic fatigue, persistent dry mouth, and marked polydipsia.

Initial laboratory evaluation revealed significant hypercalcemia, with a serum calcium level of 13.6 mg/dL (reference range: 8.5–10.3 mg/dL). PTH was detectable at 6.3 pg/mL (reference range: 10–65 pg/mL), and 25-hydroxyvitamin D was within the lower end of normal at 21.5 ng/mL (reference range: 18–50 ng/mL). Notably, 1,25-dihydroxyvitamin D was inappropriately normal at 51 pg/mL (reference range: 18–64 pg/mL), given the suppressed PTH and hypercalcemia.

**Fig. 1:**
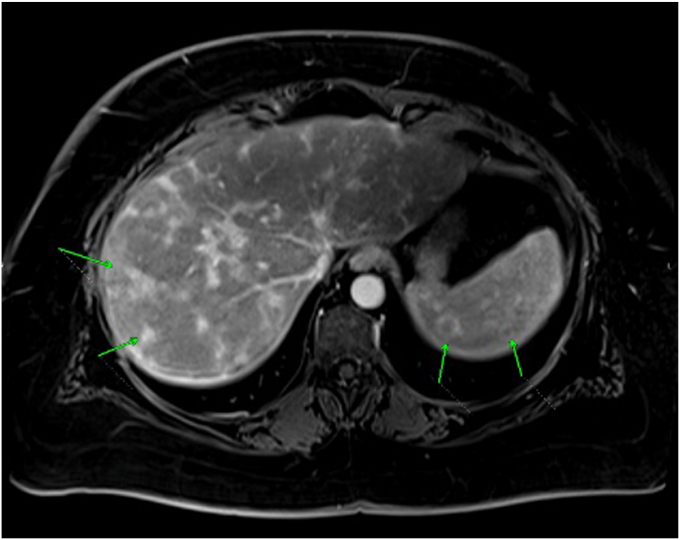
T1 axial view contrast enhanced MR image of the abdomen showing enhancing hepatic lesions and peripherally enhancing splenic lesions. *MR* = magnetic resonance.

Imaging studies were pursued to further evaluate the etiology of her symptoms and laboratory abnormalities. Computed tomography (CT) of the abdomen revealed multiple nodules in the liver and spleen, hepatic steatosis, and several mildly enlarged retroperitoneal lymph nodes. CT of the chest demonstrated an enlarged left axillary lymph node, with otherwise nonpathologically enlarged bilateral mediastinal lymph nodes. Magnetic resonance imaging of the abdomen (Fig. 1) confirmed the presence of contrast-enhancing lesions in the liver and spleen, as well as mildly enlarged retroperitoneal lymph nodes. Positron emission tomography-CT showed hypodense splenic nodules, a heterogeneous liver, diffuse pancreatic atrophy, and no evidence of abnormal adenopathy in the neck or lungs.

**Fig. 2:**
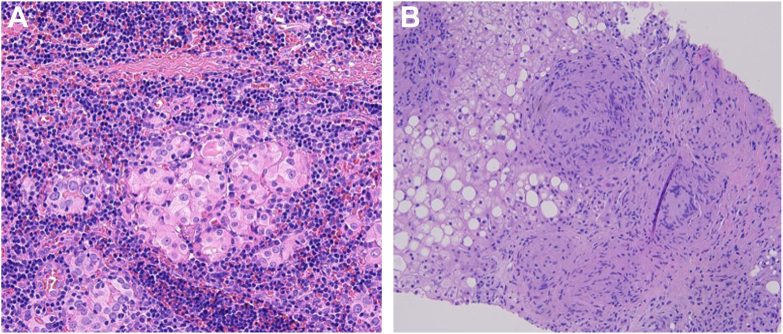
(A) Microscopic features of sarcoidosis: The granulomas are composed mostly of epithelioid histiocytes with sparse intermingled mature lymphocytes. The histiocytes show oval nuclei and abundant eosinophilic cytoplasm. (B) Granulomatous inflammation in the liver with giant cells

Given the imaging findings, a liver biopsy was performed, which revealed noncaseating granulomas (Fig. 2 *A* and *B*), consistent with a diagnosis of sarcoidosis. Further laboratory workup demonstrated an elevated angiotensin-converting enzyme (ACE) level of 124 U/L (reference range: 16-85 U/L), supporting the diagnosis.

The patient was managed acutely with calcitonin for hypercalcemia and subsequently initiated on prednisone 40 mg daily and hydroxychloroquine 800 mg daily which were both tapered down gradually. Her serum calcium levels normalized following treatment. She is currently maintained on hydroxychloroquine 200 mg and continues to do well clinically. She is followed semi-annually by both rheumatology and endocrinology for ongoing management and surveillance.

## Discussion

In patients with MEN 2A, hypercalcemia is typically due to hyperparathyroidism, making it unanticipated and rare to find hypercalcemia caused by sarcoidosis instead.

Sarcoidosis is characterized by increased extrarenal production of 1,25-dihydroxyvitamin D (calcitriol) by activated macrophages within granulomas. This leads to enhanced intestinal calcium absorption and, consequently, hypercalcemia in a subset of patients.^11^ The pathophysiology is distinct from that of primary hyperparathyroidism, which is commonly associated with MEN2A and is mediated by excessive PTH secretion.^12^ A detailed comparison of the clinical, biochemical, and management differences between sarcoidosis-related and MEN2A-associated hypercalcemia is provided in Table.

**Table:** Comparison of Hypercalcemia in MEN2A (Primary Hyperparathyroidism) and Sarcoidosis

Feature	MEN2A (primary hyperparathyroidism)	Sarcoidosis-related hypercalcemia
Pathophysiology	Excess PTH secretion from parathyroid adenoma/hyperplasia	Increased extrarenal 1,25 (OH)_2_D production by granulomas (macrophages)
Serum Calcium	Elevated	Elevated
PTH	Elevated or inappropriately normal	Suppressed
25 (OH) Vitamin D	Normal or low	Low-normal or normal
1,25 (OH)_2_ Vitamin D	Normal or mildly elevated	Inappropriately normal or elevated
ACE Level	Normal	Elevated
PTHrP	Usually not elevated	Rarely elevated; may play a minor role
Imaging	Parathyroid adenoma/hyperplasia on neck US, sestamibi, or 4D-CT	Noncaseating granulomas in affected organs (eg, lungs, liver, spleen, lymph nodes)
Histopathology	Parathyroid adenoma/hyperplasia	Noncaseating granulomas
Management	Parathyroidectomy	Corticosteroids, hydroxychloroquine, other immunosuppressants
Response to Steroids	No effect	Rapid normalization of calcium

Abbreviations: ACE = angiotensin-converting enzyme; 4D-CT = four-dimensional computed tomography; PTH = parathyroid hormone; PTHrP = parathyroid hormone-related peptide; US = ultrasound.

While in sarcoidosis, granulomas lack the negative feedback mechanism resulting in unregulated hypercalcemia and hypercalciuria.^9^^,^^13^

Elevated PTHrP levels in case reports of sarcoidosis and other granulomatous diseases along with increased PTHrP expression in bone marrow samples may indicate a role of PTHrP in the pathogenesis of hypercalcemia as well.^14^

PTHrP levels were not measured in our patient, as the suppressed PTH, absence of malignancy on imaging, and the identification of noncaseating granulomas with elevated ACE levels strongly supported sarcoidosis as the etiology of hypercalcemia, making PTHrP-mediated hypercalcemia unlikely.

In patients with both MEN2A and sarcoidosis, the risk of hypercalcemia is significantly elevated due to the convergence of these distinct mechanisms. While PHPT in MEN2A is typically managed surgically, sarcoidosis-induced hypercalcemia requires medical therapy, most commonly corticosteroids, and may also benefit from agents such as bisphosphonates or calcimimetics.

Guidelines for diagnosis and therapy of MEN stated that the indications for surgical intervention and the diagnostic criteria are similar to those in sporadic PHPT. Although often fewer than 4 parathyroid glands are enlarged, all glands should be identified at parathyroid surgery. During thyroid surgery in a normocalcemic patient with MEN2A, parathyroid tumors might be removed if there is biochemical evidence of mild hyperparathyroidism.^15^

In individuals with known genetic hypercalcemic syndromes, clinicians may initially attribute hypercalcemia to PHPT, potentially delaying the recognition of alternative or concurrent etiologies such as sarcoidosis. Optimal management in such cases necessitates a multidisciplinary and individualized approach. The diagnostic approach in this case was guided by the suppressed PTH, low-normal 25-hydroxyvitamin D, inappropriately normal 1,25-dihydroxyvitamin D, and elevated ACE levels, along with imaging and histopathological evidence of noncaseating granulomas, all of which strongly supported sarcoidosis as the etiology of hypercalcemia rather than MEN2A-related hyperparathyroidism. The patient’s hypercalcemia resolved with therapy directed at sarcoidosis, further confirming the diagnosis. If PTH levels had been normal, high-normal, or elevated, further imaging with neck ultrasound, parathyroid scintigraphy, or four-dimensional CT would have been warranted to assess for parathyroid pathology.

Regular monitoring of serum calcium and PTH levels is essential, along with appropriate imaging studies to evaluate disease activity and complications related to both MEN2A and sarcoidosis. Long-term follow-up with endocrinology and rheumatology is recommended to ensure comprehensive care and timely intervention for disease manifestations.^13^

In summary, the rare coexistence of MEN2A and sarcoidosis is exceedingly rare and requires heightened clinical vigilance, a broad diagnostic perspective, and tailored therapeutic strategies to achieve optimal patient outcomes.

## Statement of Patient Consent

Details that might identify a patient or that might enable a patient to identify him or herself were eliminated. Informed consent was obtained.

## Disclosure

The authors have no conflicts of interest to disclose.
